# Time to healthcare seeking following the onset of symptoms among men and women attending a sexual health clinic in Melbourne, Australia

**DOI:** 10.3389/fmed.2022.915399

**Published:** 2022-10-25

**Authors:** Rebecca M. Farquharson, Christopher K. Fairley, Esha Abraham, Catriona S. Bradshaw, Erica L. Plummer, Jason J. Ong, Lenka A. Vodstrcil, Marcus Y. Chen, Tiffany R. Phillips, Eric P. F. Chow

**Affiliations:** ^1^Melbourne Sexual Health Centre, The Alfred, Melbourne, VIC, Australia; ^2^Central Clinical School, Faculty of Medicine, Nursing and Health Sciences, Monash University, Melbourne, VIC, Australia; ^3^Centre for Epidemiology and Biostatistics, Melbourne School of Population and Global Health, The University of Melbourne, Melbourne, VIC, Australia

**Keywords:** sexually transmitted infection, sexually transmitted disease, sexual health, symptoms, heterosexual, men who have sex with men, healthcare, health services

## Abstract

**Background:**

Timely diagnosis and treatment of sexually transmitted infections (STIs) underpin their control by reducing the duration of infectiousness. There are currently limited data exploring healthcare seeking among individuals with STI symptoms.

**Methods:**

We analyzed data on individuals reporting STI symptoms at the Melbourne Sexual Health Centre (MSHC) between August 2017 and December 2020. We calculated the time between symptom onset and clinic attendance by risk group for 13 STI diagnoses. We performed univariable and multivariable logistic regression analyses to explore factors associated with delayed healthcare seeking (greater than 7 days).

**Results:**

Among 7,032 symptomatic clinic attendances, the shortest time to healthcare seeking was among individuals diagnosed with gonococcal urethritis (median 3 days), and the longest was among individuals diagnosed with genital warts (median 60 days). Individuals diagnosed with gonococcal urethritis sought care earlier than individuals diagnosed with non-gonococcal urethritis (median 3 vs. 6 days, *p* < 0.001), and individuals diagnosed with genital herpes sought care earlier than individuals diagnosed with primary syphilis (median 4 vs. 14 days, *p* < 0.001). Men who have sex with men, and men taking human immunodeficiency virus pre-exposure prophylaxis (PrEP), were least likely to delay healthcare seeking. Both men and women who delayed healthcare seeking were more likely to live further from the clinic than those who did not delay their presentation [*p*_*trend*_ < 0.001 (men) and *p*_*trend*_ = 0.049 (women)].

**Conclusion:**

Improved local access to healthcare alongside targeted strategies to encourage early healthcare seeking among groups at increased likelihood of delay may reduce STI-associated morbidity and transmission.

## Introduction

Notifications for sexually transmitted infections (STIs), particularly gonorrhea and syphilis, are currently rising among men and women in many countries (1, 2). Timely diagnosis and treatment of STIs underpin their control because the duration of infectiousness is reduced (3). Additionally, early treatment can reduce negative health consequences, such as infertility, chronic pain, and fetal and neonatal morbidity and mortality (4, 5).

Healthcare-seeking behavior is the process of identifying a health concern, interpreting it, deciding what course of action to take, and seeking care (6). The initial concern that precipitates healthcare seeking can vary. However, the presence of symptoms [e.g., urethral or abnormal vaginal discharge, pain, and genital warts or ulcers (7)] has been found to be the most common reason for presentation to sexual health clinics (8–10).

International studies have found that delayed healthcare seeking (defined as symptom duration greater than 7 days (11–20) following the onset of STI-associated symptoms is common (28–82% of cases) (11–20). Factors associated with delayed healthcare seeking following STI symptom onset include limited access to healthcare services (17–19), STI-associated stigma (19, 20), lack of STI-related knowledge (18, 20), and low perceived severity of symptoms (20). However, there are limited studies examining whether sexual practices or STI diagnoses affect the time to healthcare seeking. Two previous studies found that the likelihood of delayed healthcare seeking varied by diagnosis, but neither stratified individuals by risk group (15, 17). We undertook this study to examine the time to healthcare seeking following the onset of STI-associated symptoms stratified by risk group and diagnosis, and to identify risk factors associated with delayed healthcare seeking in our clinic population. Identifying specific groups or factors associated with delays in healthcare seeking enables the development of strategies to encourage earlier attendance.

## Methods

This study was a retrospective analysis of symptomatic consultations among men and women attending the Melbourne Sexual Health Centre (MSHC) between 10 August 2017 and 31 December 2020. The MSHC is the largest public sexual health service in the state of Victoria, Australia, and provides free sexual health services.

Upon arrival at the MSHC, individuals are asked to complete a questionnaire via computer-assisted self-interviewing (CASI). CASI collects data on demographic factors, sexual practices, and STI risk factors, and is used as part of routine clinical care. From 10 August 2017 onward, individuals were also asked whether they had symptoms on the day of attendance and for the duration of those symptoms (in days/weeks/months). Individuals were not asked to complete CASI if they had already done it within 3 months. Individuals could decline to answer any question on CASI. If any clinical diagnoses were made by the treating clinician, they were entered into the client’s electronic medical record. STI testing was undertaken based on clinical presentation and risk profile, as per the Australian STI management guidelines (21), and laboratory diagnoses were uploaded into the client’s electronic medical record.

We included men and women who attended the MSHC, reported symptoms and their duration via CASI between 10 August 2017 and 31 December 2020, and were clinically diagnosed with one of the 13 included STIs or genital infections. Diagnoses for women included bacterial vaginosis, candidiasis, and pelvic inflammatory disease. Diagnoses for men included gonococcal urethritis, non-gonococcal urethritis, balanitis, and proctitis. Diagnoses for both men and women included molluscum contagiosum, genital warts, anal warts, primary syphilis, secondary syphilis, and genital herpes. Clinical diagnoses were used as they are indicative of the clinical syndrome with which an individual is presenting. To ensure an accurate distinction between gonococcal and non-gonococcal urethritis, clinical diagnoses of gonococcal urethritis were confirmed with laboratory diagnosis via culture or nucleic acid amplification testing (NAAT) using the Transcription-Mediated Amplification Aptima Combo 2 Assay (Hologic Gen-Probe, San Diego, CA, USA). Individuals with a clinical diagnosis of gonococcal urethritis but a negative laboratory result for gonorrhea were excluded. Individuals given a clinical diagnosis other than gonococcal urethritis with laboratory-confirmed gonorrhea were also excluded. We excluded repeat consults within 30 days of a previous symptomatic consult and individuals with multiple clinical diagnoses in the same consult. Sex workers were excluded as during the study period there were legal requirements in Victoria to undergo 3-monthly STI testing and not work while symptomatic, which may influence healthcare-seeking behavior (22, 23).

Individuals were stratified into three groups: men who have sex with men (MSM), men who have sex with women only (MSW), and women. MSM were defined as men who had ever had sex with another man. MSW were defined as sexually active men who had never had sex with another man. Women were not stratified based on sexual practices due to the small number of women who have sex with women attending the MSHC (24). We calculated the straight-line distance between the individual’s residential postcode and the MSHC, to determine the distance from home to the MSHC. Socioeconomic status (SES) quintile by postcode was determined using the Index of Relative Socio-economic Advantage and Disadvantage (IRSAD) 2016 (25). Condomless sex was defined as individuals who reported that they did not always use condoms during vaginal or anal sex in the past 3 months. Time to healthcare seeking was defined as the reported duration of symptoms on CASI and expressed in days. Delayed healthcare seeking was defined as individuals who reported a symptom duration of more than 7 days (11–20).

Descriptive statistics and frequencies were calculated. Time to healthcare seeking in days was compared between MSM, MSW, and women for each diagnosis. Mann–Whitney U tests were used for comparisons between two groups (i.e., within diagnoses only seen in men), and Kruskal–Wallis H tests were used for comparisons between three groups. Pairwise comparisons using *post-hoc* Dunn tests with Bonferroni correction were conducted where results were significant on Kruskal–Wallis H test. Time to healthcare seeking in days was also compared between diagnoses with comparable clinical presentations (i.e., gonococcal urethritis and non-gonococcal urethritis, bacterial vaginosis and candidiasis, primary syphilis and genital herpes, and genital warts and anal warts) using Mann–Whitney U tests and illustrated using a violin plot.

Univariable logistic regression with a generalized estimating equation (GEE) using the exchangeable correlation structure, clustered by diagnosis, was performed to determine associations between delayed healthcare seeking and possible explanatory variables. Multivariable GEE logistic regression analyses were subsequently performed, adjusting for factors with p < 0.10 in the univariable analysis. Crude and adjusted odds ratios (aOR) and 95% confidence intervals (CI) are reported. *p*-Values for linear trend (*p*_*trend*_ values) for ordinal variables were also calculated using GEE logistic regression, clustered by diagnosis. Logistic regression analyses were performed separately for men and women, as some STIs applied to either men or women only.

All statistical analyses were conducted using Stata version 17 (StataCorp, College Station, TX, USA). Ethics approval for this study was obtained from the Alfred Hospital Ethics Committee, Melbourne, Australia (247/20).

## Results

**FIGURE 1:**
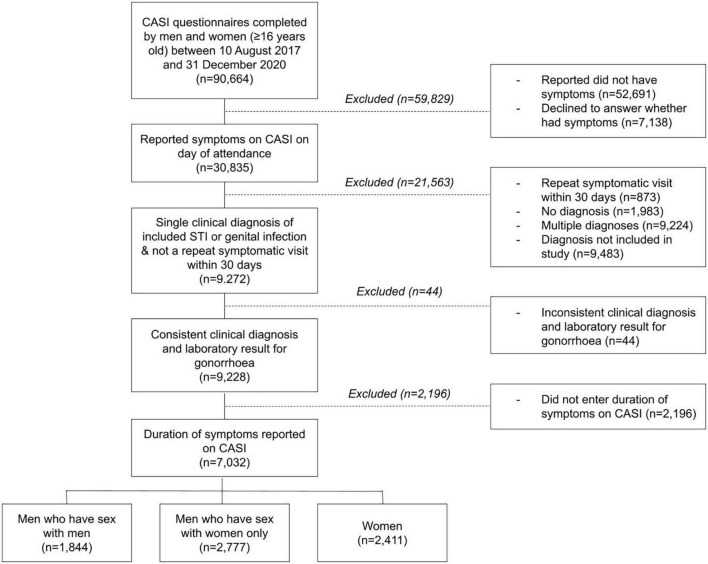
Flowchart of the sample selection process. Individuals with one of the following 13 clinical diagnoses were eligible for inclusion: bacterial vaginosis, candidiasis, pelvic inflammatory disease, gonococcal urethritis, non-gonococcal urethritis, balanitis, molluscum contagiosum, proctitis, genital warts, anal warts, primary syphilis, secondary syphilis, and genital herpes.

Between 10 August 2017 and 31 December 2020, 90,664 men and women completed the questionnaire via CASI (Figure 1). Of those, 30,835 individuals reported symptoms, 52,691 individuals did not have symptoms, and 7,138 declined to answer whether they had symptoms. Of the 30,835 individuals who reported symptoms, 9,272 (30.1%) were eligible for inclusion, as they had one and only one of the included STIs or genital infections and were not attending a repeat clinic visit within 30 days of previous symptomatic attendance. We further excluded 44 individuals with inconsistent clinical diagnosis and laboratory test results for gonorrhea. Of the remaining 9,228 individuals, 7,032 reported the duration of their symptoms via CASI and were included in the final analysis. The median age of included individuals was 29 years [interquartile range (IQR) 25–35]. There were 2,411 (34.3%) women, 1,844 (26.2%) MSM, and 2,777 (39.5%) MSW. The most common diagnosis was non-gonococcal urethritis in men [53.5% (*n* = 896) in MSM and 38.9% (*n* = 1,069) in MSW], and candidiasis in women [42.9% (*n* = 1027)].

### Time to healthcare seeking by risk group

The MSM group sought care earlier than the MSW group among individuals diagnosed with non-gonococcal urethritis (median 5 vs. 7 days, *p* < 0.001), genital warts (median 30 vs. 60 days, *p* = 0.001), and anal warts (median 30 vs. 150 days, *p* < 0.001) (Table 1). Among individuals diagnosed with genital warts, women sought care earlier than MSW (median 30 vs. 60 days, *p* = 0.002). Among individuals diagnosed with molluscum contagiosum, women sought care later than both MSM (median 42 vs. 14 days, *p* = 0.003) and MSW (median 42 vs. 30 days, *p* = 0.048).

**TABLE 1:** Time to healthcare seeking following symptom onset in days by risk group, by diagnosis.

Diagnosis	MSM		MSW		Women		Significance			
	*N*	Median days (IQR)	*N*	Median days (IQR)	*N*	Median days (IQR)	*P*-value* (MSM, MSW and women)	*P*-value^†^ (MSM and MSW)	*P*-value^†^ (MSM and women)	*P*-value^†^ (MSW and women)
Bacterial Vaginosis	–	–	–	–	775	14 (5–28)	–	–	–	–
Candidiasis	–	–	–	–	1027	7 (3–14)	–	–	–	–
Pelvic Inflammatory Disease	–	–	–	–	166	14 (5–28)	–	–	–	–
Gonococcal Urethritis	112	3 (2–4)	96	3 (2–5)	–	–	–	0.320	–	–
Non-Gonococcal Urethritis	896	5 (2–7)	1069	7 (3–14)	–	–	–	<0.001	–	–
Balanitis	200	7 (3–21)	654	7 (3–30)	–	–	–	0.137	–	–
Proctitis^‡^	148	7 (3–14)	2	60 (60–60)	–	–	–	–	–	–
Molluscum Contagiosum	35	14 (7–35)	213	30 (7–90)	85	42 (14–90)	0.006	0.089	0.003	0.048
Genital warts	84	30 (14–60)	603	60 (21–180)	200	30 (14–105)	<0.001	0.001	0.449	0.002
Anal warts	168	30 (14–60)	32	150 (45–225)	16	75 (21–165)	<0.001	<0.001	0.086	0.225
Genital herpes	84	4 (2–7)	88	4 (2–7)	133	4 (2–7)	0.359	–	–	–
Primary syphilis	70	7 (4–21)	11	14 (7–30)	4	21 (14–74)	0.086	–	–	–
Secondary syphilis	47	14 (5–21)	9	21 (14–30)	5	21 (14–90)	0.188	–	–	–

IQR, interquartile range; MSM, men who have sex with men; MSW, men who have sex with women only; N, the total number of individuals with the diagnosis.

*P-values were calculated with Kruskal–Wallis H test.

†*P*-values were calculated with Mann-Whitney U test (gonococcal urethritis, non-gonococcal urethritis, balanitis) or with post hoc Dunn test (molluscum contagiosum, genital warts, anal warts).

^‡^Tests of comparison were not conducted for proctitis as there were only two MSW with proctitis.

Bacterial vaginosis (median 14 days, IQR = 5–28), candidiasis (median = 7 days, IQR = 3–14), and pelvic inflammatory disease (PID) (median = 14 days, IQR = 5–28) were analyzed only in women. Among individuals diagnosed with gonococcal urethritis, balanitis, genital herpes, primary syphilis, and secondary syphilis, there were no differences in time to healthcare seeking by risk group (Table 1).

### Time to healthcare seeking by diagnosis

The shortest time to healthcare seeking was among individuals diagnosed with gonococcal urethritis (median = 3 days, IQR = 2–5). The longest time to healthcare seeking was among individuals diagnosed with genital warts (median = 60 days, IQR = 14–150).

**FIGURE 2:**
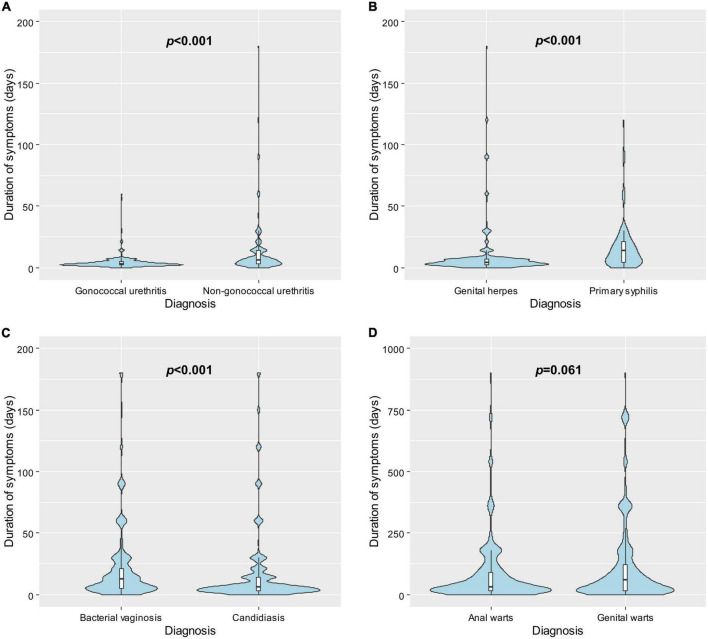
Violin plots showing the distributions of the time to healthcare seeking following symptom onset in days by diagnosis and box plots showing the observations from the 25th to the 75th percentiles among individuals with **(A)** gonococcal urethritis vs. non-gonococcal urethritis; **(B)** genital herpes vs. primary syphilis; **(C)** bacterial vaginosis vs. candidiasis; and **(D)** anal warts vs. genital warts. *P*-values were calculated using Mann–Whitney U test.

Several differences in time to healthcare seeking between comparable diagnoses were identified (Figure 2). Individuals diagnosed with gonococcal urethritis sought care earlier than individuals diagnosed with non-gonococcal urethritis (median 3 vs. 6 days, *p* < 0.001). Individuals diagnosed with candidiasis sought care earlier than individuals diagnosed with bacterial vaginosis (median 7 vs. 14 days, *p* < 0.001). Furthermore, individuals diagnosed with genital herpes sought care earlier than individuals diagnosed with primary syphilis (median 4 vs. 14 days, *p* < 0.001). There was no significant difference in time to healthcare seeking between individuals diagnosed with genital warts and individuals diagnosed with anal warts (median 30 vs. 60 days, *p* = 0.061).

### Delayed healthcare seeking

Within our sample, 34.5% (637/1,844) of MSM, 52.5% (1,457/2,777) of MSW, and 45.9% (1,106/2,411) of women delayed healthcare seeking for more than 7 days.

The MSM group was less likely to delay healthcare seeking than the MSW group (aOR = 0.74; 95% CI = 0.67–0.81). Additionally, an increasing number of male partners and an increasing number of female partners in the past 3 months were both associated with decreased odds of delayed healthcare seeking among men (*p*_*trend*_ = 0.001 and *p*_*trend*_ = 0.009, respectively) (Table 2). An increasing number of male partners in the past 3 months was also associated with decreased odds of delayed healthcare among women (*p*_*trend*_ = 0.001) (Table 3). HIV-negative men taking PrEP were less likely to delay healthcare seeking than HIV-negative men not taking PrEP (aOR = 0.64; 95% CI = 0.57–0.73). However, there was no difference in delayed healthcare seeking between men living with HIV and men not living with HIV (*p* = 0.103). In women, HIV status was not associated with delayed healthcare seeking (Table 3).

**TABLE 2:** Factors associated with delayed healthcare seeking (>7 days) among 4,621 symptomatic men attending the Melbourne Sexual Health Centre, 10 August 2017 to 31 December 2020.

	Early healthcare seeking, *n* (%) *N* = 2,527 (100%)*	Delayed healthcare seeking, *n* (%) *N* = 2,094 (100%)*	Proportion of individuals with delayed healthcare seeking (%)^†^	Crude OR^‡^ (95% CI)	*P*-value	Adjusted OR^‡^ (95% CI)	*P*-value
**Risk group**							
MSW	1,320 (52.2)	1,457 (69.6)	52.5	1.00 (ref)		1.00 (ref)	
MSM	1,207 (47.8)	637 (30.4)	34.5	0.61 (0.51–0.73)	<0.001	0.74 (0.67–0.81)	<0.001
**Age (years)**							
16–24	400 (15.8)	343 (16.4)	46.2	1.00 (ref)		–	–
25–34	1,226 (48.5)	1,032 (49.3)	45.7	1.03 (0.79–1.36)	0.811	–	–
≥35	901 (35.7)	719 (34.3)	44.4	1.18 (0.87–1.60)	0.284	–	–
**Distance from home to the clinic ^¶^**					*p*_*trend*_ < 0.001**		*p*_*trend*_ < 0.001**
<5 km	889 (35.2)	596 (28.5)	40.1	1.00 (ref)		1.00 (ref)	
5–15 km	704 (27.9)	615 (29.4)	46.6	1.24 (1.13–1.35)	<0.001	1.20 (1.08–1.32)	<0.001
15–25 km	219 (8.7)	189 (9.0)	46.3	1.35 (1.17–1.56)	<0.001	1.27 (1.11–1.46)	0.001
25–50 km	110 (4.4)	133 (6.4)	54.7	1.66 (1.40–1.98)	<0.001	1.56 (1.31–1.85)	<0.001
>50 km	64 (2.5)	77 (3.7)	54.6	1.54 (1.21–1.97)	0.001	1.46 (1.14–1.87)	0.003
Interstate	27 (1.1)	16 (0.8)	37.2	1.22 (0.86–1.74)	0.268	1.18 (0.81–1.73)	0.395
Unknown	514 (20.3)	468 (22.3)	47.7	1.32 (1.20–1.46)	<0.001	1.24 (1.11–1.38)	<0.001
**Country of birth**							
Australia/New Zealand	1,395 (55.2)	1,132 (54.1)	44.8	1.00 (ref)		1.00 (ref)	
Overseas	1,058 (41.9)	890 (42.5)	45.7	0.98 (0.84–1.15)	0.842	0.99 (0.85–1.15)	0.910
Unknown	74 (2.9)	72 (3.4)	49.3	1.22 (1.01–1.47)	0.038	1.17 (0.97–1.41)	0.091
**Previous STI (self-reported)**							
No	903 (35.7)	766 (36.6)	45.9	1.00 (ref)		1.00 (ref)	
Yes	1,494 (59.1)	1,154 (55.1)	43.6	0.98 (0.87–1.12)	0.810	1.16 (1.04–1.28)	0.005
Unknown	130 (5.1)	174 (8.3)	57.2	1.31 (1.05–1.65)	0.019	1.35 (1.04–1.76)	0.026
**Injecting drug use in the past 3 months**							
No	2,480 (98.1)	2,051 (97.9)	45.3	1.00 (ref)		1.00 (ref)	
Yes	28 (1.1)	20 (1.0)	41.7	1.04 (0.67–1.63)	0.854	1.17 (0.74–1.85)	0.510
Unknown	19 (0.8)	23 (1.1)	54.8	1.55 (1.17–2.05)	0.002	1.70 (1.26–2.28)	<0.001
**Number of male partners in the past 3 months**					*p*_*trend*_ < 0.001		*p*_*trend*_ = 0.001
0	1,472 (58.3)	1,569 (74.9)	51.6	1.00 (ref)		1.00 (ref)	
1	226 (8.9)	183 (8.7)	44.7	0.79 (0.68–0.91)	0.001	1.11 (0.87–1.42)	0.408
2–3	339 (13.4)	164 (7.8)	32.6	0.59 (0.46–0.77)	<0.001	0.88 (0.68–1.14)	0.343
≥4	490 (19.4)	178 (8.5)	26.6	0.51 (0.39–0.66)	<0.001	0.83 (0.61–1.12)	0.219
**Number of female partners in the past 3 months**					*p*_*trend*_ = 0.353		*p*_*trend*_ = 0.009
0	1,093 (43.3)	681 (32.5)	38.4	1.00 (ref)		1.00 (ref)	
1	466 (18.4)	652 (31.1)	58.3	1.63 (1.14–2.35)	0.008	1.25 (0.92–1.70)	0.157
2–3	627 (24.8)	521 (24.9)	45.4	1.33 (0.99–1.79)	0.062	0.98 (0.76–1.27)	0.879
≥4	341 (13.5)	240 (11.5)	41.3	1.20 (0.78–1.85)	0.402	0.89 (0.66–1.20)	0.463
**Condomless sex in the past 3 months**							
No	393 (15.6)	348 (16.6)	47.0	1.00 (ref)		1.00 (ref)	
Yes	1,950 (77.2)	1,482 (70.8)	43.2	1.01 (0.87–1.16)	0.917	1.02 (0.90–1.14)	0.770
N/A (no sexual partners)	41 (1.6)	124 (5.9)	75.2	1.76 (1.27–2.44)	0.001	1.75 (1.42–2.17)	<0.001
N/A (no vaginal/anal sex with partners)	105 (4.2)	108 (5.2)	50.7	1.26 (0.91–1.76)	0.169	1.25 (0.95–1.65)	0.109
Unknown	38 (1.5)	32 (1.5)	45.7	1.02 (0.68–1.52)	0.929	0.97 (0.61–1.52)	0.882
**HIV status**							
HIV negative not taking PrEP	2,072 (82.0)	1,937 (92.5)	48.3	1.00 (ref)		1.00 (ref)	
HIV negative taking PrEP	357 (14.1)	114 (5.4)	24.2	0.48 (0.38–0.60)	<0.001	0.64 (0.57–0.73)	<0.001
HIV positive	98 (3.9)	43 (2.1)	30.5	0.73 (0.61–0.87)	0.001	0.84 (0.68–1.04)	0.103
**Socioeconomic status quintile (Postcode)^†⁣†^**							
1	104 (4.1)	83 (4.0)	44.4	1.00 (ref)		–	–
2	99 (3.9)	79 (3.8)	44.4	1.04 (0.83–1.29)	0.756	–	–
3	183 (7.2)	149 (7.1)	44.9	0.99 (0.64–1.53)	0.958	–	–
4	510 (20.2)	475 (22.7)	48.2	1.06 (0.83–1.35)	0.627	–	–
5	1,089 (43.1)	824 (39.4)	43.1	0.91 (0.72–1.14)	0.404	–	–
Interstate	27 (1.1)	16 (0.8)	37.2	1.00 (0.88–1.32)	0.993	–	–
Unknown	515 (20.4)	468 (22.3)	47.6	1.08 (0.88–1.32)	0.447	–	–

n represents the number of individuals in each category; MSM represents men who have sex with men; MSW represents men who have sex with women only; N/A represents not applicable; HIV represents human immunodeficiency virus; PrEP represents pre-exposure prophylaxis; OR represents odds ratio.

*Column percentages were presented for the proportion of individuals in each category.

^†^Row percentages were presented for the proportion of individuals with delayed healthcare seeking.

^‡^Effect estimates derived from a generalised estimated equation logistic regression model, clustered by diagnosis.

^¶^ Straight-line distance from the individual’s residential postcode to the Melbourne Sexual Health Centre.

**p_trend_ was calculated by excluding the “interstate” and “unknown postcode” categories.

^††^Socioeconomic status (SES) quintile by postcode was determined using the Index of Relative Socio-economic Advantage and Disadvantage (IRSAD) 2016.

**TABLE 3:** Factors associated with delayed healthcare seeking (>7 days) among 2,411 symptomatic women attending the Melbourne Sexual Health Centre, 10 August 2017 to 31 December 2020.

	Early healthcare seeking, *n* (%) *N* = 1,305 (100%)*	Delayed healthcare seeking, *n* (%) *N* = 1,106 (100%)*	Proportion of individuals with delayed healthcare seeking (%)^†^	Crude OR^‡^ (95% CI)	*P*-value	Adjusted OR^‡^ (95% CI)	*P*-value
**Age (years)**							
16–24	422 (32.3)	332 (30.0)	44.0	1.00 (ref)		–	–
25–34	733 (56.2)	628 (56.8)	46.1	1.06 (0.94–1.18)	0.352	–	–
≥35	150 (11.5)	146 (13.2)	49.3	1.07 (0.91–1.26)	0.410	–	–
**Distance from home to the clinic ^¶^**					*p*_*trend*_ = 0.019**		*p*_*trend*_ = 0.049**
<5 km	574 (44.0)	440 (39.8)	43.4	1.00 (ref)		1.00 (ref)	
5–15 km	344 (26.4)	322 (29.1)	48.3	1.14 (1.00–1.29)	0.043	1.13 (1.00–1.30)	0.053
15–25 km	48 (3.7)	60 (5.4)	55.6	1.48 (1.15–1.92)	0.003	1.51 (1.15–1.98)	0.003
25–50 km	36 (2.8)	29 (2.6)	44.6	0.79 (0.54–1.14)	0.210	0.77 (0.50–1.19)	0.242
>50 km	11 (0.8)	20 (1.8)	64.5	2.29 (0.84–6.28)	0.106	2.19 (0.81–5.93)	0.123
Interstate	8 (0.6)	3 (0.3)	27.3	0.62 (0.23–1.69)	0.354	0.67 (0.23–1.95)	0.463
Unknown	284 (21.8)	232 (21.0)	45.0	1.10 (0.89–1.36)	0.389	1.10 (0.87–1.38)	0.440
**Country of birth**							
Australia/New Zealand	443 (33.9)	388 (35.1)	46.7	1.00 (ref)		–	–
Overseas	818 (62.7)	696 (62.9)	46.0	0.93 (0.81–1.06)	0.285	–	–
Unknown	44 (3.4)	22 (2.0)	33.3	0.66 (0.40–1.09)	0.102	–	–
**Previous STI (self-reported)**							
No	672 (51.5)	499 (45.1)	42.6	1.00 (ref)		–	–
Yes	546 (41.8)	517 (46.7)	48.6	1.07 (0.93–1.23)	0.341	–	–
Unknown	87 (6.7)	90 (8.1)	50.8	1.09 (0.66–1.82)	0.732	–	–
**Injecting drug use in the past 3 months**							
No	1,289 (98.8)	1,090 (98.6)	45.8	1.00 (ref)		–	–
Yes	2 (0.2)	6 (0.5)	75.0	2.99 (0.50–17.73)	0.228	–	–
Unknown	14 (1.1)	10 (0.9)	41.7	0.82 (0.63–1.07)	0.145	–	–
**Number of male partners in the past 3 months**					*p*_*trend*_ < 0.001		*p*_*trend*_ = 0.001
0	68 (5.2)	125 (11.3)	64.8	1.00 (ref)		1.00 (ref)	
1	448 (34.3)	433 (39.2)	49.1	0.62 (0.43–0.91)	0.015	0.88 (0.58–1.35)	0.565
2–3	596 (45.7)	424 (38.3)	41.6	0.48 (0.38–0.61)	< 0.001	0.69 (0.46–1.02)	0.064
≥4	193 (14.8)	124 (11.2)	39.1	0.45 (0.29–0.70)	< 0.001	0.64 (0.37–1.09)	0.099
**Number of female partners in the past 3 months**					*p*_*trend*_ = 0.190		*p*_*trend*_ = 0.484
0	1,183 (90.7)	988 (89.3)	45.5	1.00 (ref)		1.00 (ref)	
1	83 (6.4)	70 (6.3)	45.8	1.08 (0.90–1.29)	0.624	1.00 (0.88–1.14)	0.961
2–3	30 (2.3)	43 (3.9)	58.9	2.24 (1.52–3.30)	0.010	1.41 (0.81–2.46)	0.227
≥ 4	9 (0.7)	5 (0.5)	35.7	0.72 (0.31–1.67)	0.440	0.76 (0.33–1.72)	0.510
**Condomless sex in the past 3 months**							
No	165 (12.6)	119 (10.8)	41.9	1.00 (ref)		1.00 (ref)	
Yes	1,062 (81.4)	856 (77.4)	44.6	1.08 (0.90–1.29)	0.418	1.13 (0.91–1.40)	0.267
N/A (no sexual partners)	22 (1.7)	52 (4.7)	70.3	2.24 (1.52–3.30)	< 0.001	1.78 (0.82–3.86)	0.145
N/A (no vaginal/anal sex with partners)	37 (2.8)	63 (5.7)	63.0	2.02 (1.38–2.96)	< 0.001	1.54 (1.19–1.99)	0.001
Unknown	19 (1.5)	16 (1.4)	45.7	1.30 (0.67–2.51)	0.440	1.13 (0.66–1.94)	0.658
**HIV status^††^**							
HIV negative	1,302 (99.8)	1,105 (99.9)	45.9	1.00 (ref)		–	–
HIV positive	3 (0.2)	1 (0.1)	25.0	0.49 (0.09–2.62)	0.407	–	–
**Socioeconomic status quintile (Postcode)^‡‡^**							
1	27 (2.1)	32 (2.9)	54.2	1.00 (ref)		–	–
2	20 (1.5)	25 (2.3)	55.6	1.05 (0.69–1.57)	0.831	–	–
3	82 (6.3)	56 (5.1)	40.6	0.67 (0.37–1.22)	0.188	–	–
4	299 (22.9)	225 (20.3)	42.3	0.70 (0.50–1.00)	0.050	–	–
5	585 (44.8)	533 (48.2)	47.7	0.90 (0.58–1.38)	0.627	–	–
Interstate	8 (0.6)	3 (0.3)	27.3	0.48 (0.16–1.44)	0.190	–	–
Unknown	284 (21.8)	232 (21.0)	45.0	0.85 (0.60–1.20)	0.352	–	–

Note. n represents the number of individuals in each category; N/A represents not applicable; HIV represents human immunodeficiency virus; OR represents odds ratio.

*Column percentages were presented for the proportion of individuals in each category.

^†^Row percentages were presented for the proportion of individuals with delayed healthcare seeking.

^‡^Effect estimates derived from a generalized estimated equation logistic regression model, clustered by diagnosis.

^¶^ Straight-line distance from the individual’s residential postcode to the Melbourne Sexual Health Centre.

**p_trend_ was calculated by excluding the “interstate” and “unknown postcode” categories.

^††^No women in the study population reported taking HIV pre-exposure prophylaxis.

^‡‡^Socioeconomic status (SES) quintile by postcode was determined using the Index of Relative Socio-economic Advantage and Disadvantage (IRSAD) 2016.

Among both men and women, increasing distance from home to the clinic was significantly associated with an increased likelihood of delayed healthcare seeking [*p*_*trend*_ < 0.001 (men) and *p*_*trend*_ = 0.049 (women)]. Other demographic factors including socioeconomic status by postcode, age group, and country of birth did not affect the odds of delayed healthcare seeking among men or women (Tables 2, 3).

## Discussion

In this study, we found that MSM sought healthcare earlier than MSW, and that individuals diagnosed with gonococcal urethritis and genital herpes, which are typically associated with marked symptoms including purulent discharge and pain, presented earlier than patients diagnosed with non-gonococcal urethritis and primary syphilis, which typically cause milder symptoms (26, 27). We also found that men and women living further than 5 km from the clinic delayed health care seeking more than men and women living within 5 km of the clinic. Our study is one of the largest to date examining time to healthcare seeking among individuals with STI-associated symptoms. We extend the findings of Hook et al. and Liu et al., both of whom found that the likelihood of delayed healthcare seeking differed across 6–9 STI diagnoses (15, 17), by demonstrating differences in time to healthcare seeking across three at-risk populations and 13 STIs and genital infections. Our data provide potential opportunities for targeted interventions to encourage early healthcare seeking following the onset of STI-associated symptoms, including positioning of clinical services that provide ready access to local populations.

One important finding in our study was that as the distance from home to the clinic increased, the likelihood of delayed healthcare seeking also increased, among both men and women. This is consistent with previous studies internationally. Thi Thu et al. found that Vietnamese women living in rural or remote areas had a longer time to healthcare seeking at a metropolitan STI clinic than those living in urban areas (18). Puthuchira Ravi et al. found that women in rural India who lived within 1 km of a healthcare facility were more likely to seek healthcare for their STI-associated symptoms than those who lived further away (28). Our study builds on and extends these past findings by reporting a progressive increase in the likelihood of delayed healthcare seeking among men and women as the distance from a metropolitan sexual health service increases. Our finding is of particular concern because outer Melbourne suburbs have high notification rates of gonorrhea and syphilis, particularly among women (29, 30). In addition, our finding that SES by postcode was not associated with a change in the likelihood of delay suggests that geographical distance itself may play a role, rather than being solely reflective of social disadvantage. The MSHC is the only public HIV/STI clinic in Melbourne, a city of more than 5 million people (31), and it is likely the only feasible option for free sexual health care for many Melbournians, particularly those without a Medicare card (i.e., Australia’s universal health insurance scheme) and those who feel uncomfortable seeing their local general practitioner.

We found that MSM sought healthcare earlier than MSW among individuals with non-gonococcal urethritis, genital warts, and anal warts. This finding builds on one previous Melbourne-based study on urethral gonorrhea suggesting that time to healthcare seeking was shorter among MSM than MSW (32). Several factors may explain our findings. Earlier healthcare seeking among MSM, and among other high-risk groups including men taking PrEP, may relate to directed targeting of existing public health initiatives (33), the recommendation for regular STI screening among MSM in Australia (21), or high perceived STI risk among MSM (34). However, what is inconsistent with this is our finding that the differences in time to healthcare seeking between risk groups were present only in a select group of diagnoses. Our results suggest that time to healthcare seeking differs by risk group predominantly among individuals with less acute STIs, including genital warts and anal warts, and not among individuals with more acute STIs, including gonococcal urethritis and genital herpes. It may be hypothesized that when symptoms are less disruptive, other factors play a larger role in determining the time to healthcare seeking.

We found that the diagnosis with the longest time to healthcare seeking was genital warts (median 60 days), and the diagnosis with the shortest time to healthcare seeking was gonococcal urethritis (median 3 days). These findings echo those of Hook et al. and Liu et al., both of whom found that men with gonorrhea were the least likely to delay healthcare seeking (15, 17), with Hook et al. additionally finding that men and women with genital warts were the most likely to delay (15). We build on this existing literature by quantifying symptom duration in days. Furthermore, we identified multiple differences in time to healthcare seeking between comparable diagnoses. We found that men diagnosed with gonococcal urethritis sought care earlier than men diagnosed with non-gonococcal urethritis, echoing the findings of Hook et al. and Aaron et al. (11, 15). We also found that individuals diagnosed with genital herpes presented earlier than individuals diagnosed with primary syphilis. Individuals in our study were not asked what specific symptoms they experienced, and we cannot determine why differences in time to healthcare seeking between diagnoses occurred. However, these differences may relate to different severity of symptoms, as conditions such as gonococcal urethritis and genital herpes are typically associated with more marked symptoms compared to non-gonococcal urethritis and primary syphilis (26, 27) which are typically milder.

The proportion of individuals who delayed healthcare seeking in our study was similar to other international studies. We found that delayed healthcare seeking (more than 7 days) following symptom onset was common, seen in 46% of women, 35% of MSM, and 53% of MSW. These proportions are similar to those identified in studies in New Zealand (13), the UK (35), and the US (11, 15, 19), i.e., 38–47% of women and 28–44% of men were found to delay healthcare seeking for more than 7 days. Our findings build upon the existing literature by demonstrating that while delayed healthcare seeking was common across all groups, there was a substantial difference in the proportion of delay between MSM and MSW.

This study has several limitations. First, it was conducted at a single sexual health centre with a generally high-risk patient population. Hence, our findings may not be generalizable to other settings where people may present with STI-associated symptoms. Second, recall bias and social desirability bias regarding the self-reported duration of symptoms and other sexual practices may have occurred. Third, individuals were not asked about specific symptoms experienced, and thus we cannot comment on the effect of specific symptoms on the time to healthcare seeking. Fourth, this study included a period during the COVID-19 pandemic, and there were two major lockdowns in Melbourne in 2020 (i.e., a total of 154 days). The MSHC remained open throughout the COVID-19 pandemic (36). These two lockdowns included stay-at-home orders; however, individuals were allowed to leave home due to medical reasons, and there were no significant changes in time to healthcare seeking in Melbourne during the COVID-19 pandemic in 2020 (37). Fifth, delayed healthcare seeking was defined as seeking care more than 7 days following symptom onset, to allow for comparison to existing studies, and this cut-off may not be suitable for all diagnoses. Finally, we did not include all individuals with symptoms in the analysis and if those who were excluded (e.g., those with two or more diagnoses, and those who identify as trans or gender diverse) were systematically different, then our results may be biased.

To conclude, we found that time to healthcare seeking varied by STI diagnosis and by risk group. This highlights an opportunity for targeted health promotion to encourage early healthcare seeking among groups that are more likely to delay. By reducing the interval between symptom onset and healthcare service attendance, the duration of infectiousness of STIs could be reduced. Our findings further indicate that improved local access to healthcare, such as through additional public STI clinics, could have a substantial impact on time to healthcare seeking among individuals with STI-associated symptoms.

## Data availability statement

The datasets presented in this article are not readily available because data cannot be made publicly available in order to protect patient privacy as per the approved ethics requirement. Requests to access the datasets should be directed to EC, eric.chow@monash.edu.

## Ethics statement

The studies involving human participants were reviewed and approved by Alfred Hospital Ethics Committee, Melbourne, Australia (Project number: 247/20). Written informed consent from the participants’ legal guardian/next of kin was not required to participate in this study in accordance with the national legislation and the institutional requirements.

## Author contributions

CF, TP, and EC conceived and designed the study. RF performed the literature review, conducted the data analysis, and wrote the first draft of the manuscript. TP and EC provided the statistical advice and supervision of the project. EC assisted with data illustration and presentation. All authors were involved in data interpretation, revising the manuscript for important intellectual content, and approving the final version.
